# Rivaroxaban improves hidden blood loss, blood transfusion rate and reduces swelling of the knee joint in knee osteoarthritis patients after total knee replacement

**DOI:** 10.1097/MD.0000000000012630

**Published:** 2018-10-05

**Authors:** Yi-Min Zhang, Jian-Yong Liu, Xue-Dong Sun, Miao Zhang, Xiao-Guang Liu, Xiu-Li Chen

**Affiliations:** Department of Joint Surgery, Weifang People's Hospital, Weifang, P.R. China.

**Keywords:** blood coagulation function, blood transfusion rate, complications, dominant blood loss, hidden blood loss, hospital for special surgery, rivaroxaban, total knee replacement

## Abstract

Osteoarthritis (OA) is the third most common diagnosis made by general practitioners in older patients. The purpose of the current study is to investigate effects rivaroxaban had on both hidden blood loss and blood transfusion rate (BTR) in patients with knee OA (KOA) after going through a total knee replacement (TKR).

Between the time periods of December 2011 up until January 2015, a total of 235 patients underwent TKR and were selected to be assigned to either the rivaroxaban or nonanticoagulant groups. Coagulation function indexes before surgery and following administration of rivaroxaban, total blood loss, hidden blood loss, dominant blood loss, blood transfusion volume, hemoglobin reduction, degree of postoperative pain (visual analogue scale), the degree of knee swelling, and range of motion following surgery were all recorded. Hospital for special surgery (HSS) scores offered an objective evaluation for the knee joint functions before surgery at the intervals of 2 weeks and after surgery at intervals of 3 months, 6 months, 12 months, and 24 months.

Patients in the rivaroxaban group had shown a higher hidden blood loss, as well as a higher BTR, compared to those involved in the nonanticoagulant group. BTR was found to have been 49.59% in the rivaroxaban group, and 35.09% for the nonanticoagulant group. Patients in the rivaroxaban group had lower degrees of knee swelling than those involved in the nonanticoagulant group. There was no deep vein thrombosis (DVT) detected in the rivaroxaban group, whereas 5 DVT cases were detected in the nonanticoagulant group. In the rivaroxaban group, the HSS scores of the knee joint functions were remarkably higher at the 2-week mark in succession to the surgery than those involved with the nonanticoagulant group.

This overall data demonstrated that KOA patients after TKR had presented with a higher hidden blood loss, BRT, and lower swelling degrees of the knee joint after being treated by the rivaroxaban.

## 1 Introduction

Osteoarthritis (OA) is known as the most frequent form of arthritis, with knee OA (KOA) being the leading etiology for lower extremity disability found in older adults.^[1]^ Total knee replacement (TKR) is similarly known as the most effective orthopedic treatment for patients diagnosed with KOA, and recent reports have suggested and stressed the increasing use of TKR in the last 10 years with its satisfaction rates showing success in more than 90% of those treated.^[2,3]^ Patients 65 years or younger have shown a better chance for receiving TKR because of the high physical demands.^[4]^ TKR could relieve both pain and improve knee joint function for many young patients diagnosed with rheumatoid arthritis, proving to be a successful treatment due to its good medium-term outcomes.^[5]^ Because of both the genetic and dietary differences, there is a strikingly low prevalence of deep vein thrombosis (DVT), as well as fatal pulmonary embolism (PE), following the TKR procedure in Asian patients.^[6]^ Studies indicated that both DVT and PE were ranging between 40% and 85%, and 0.1% and 0.7%, respectively, for patients who have undergone TKR without using an anticoagulant prophylaxis.^[7,8]^ Therefore, the risk for thromboembolism can potentially be reduced by implementing the use of a pharmacological thromboprophylaxis following TKR in which there were some new oral anticoagulants, such as both the direct thrombin inhibitors, as well as factor Xa inhibitors.^[9]^

Rivaroxaban, an oral anticoagulant, has been known to not only inhibit factor Xa both directly and selectively, but also treats DVT following TKR in adult patients.^[10,11]^ It has also been reported that the utilization of rivaroxaban needs no laboratory monitoring and has no food interactions, but does present with a few drug interactions.^[12]^ Previous evidence has also revealed that rivaroxaban has been shown to prevent both venous thromboembolism (VTE) and PE, as well as decreases the incidence of stroke in atrial fibrillation patients, being both safe and effective.^[13,14]^ A previous study indicated that a great amount of blood was lost in the process of total joint replacement and about 2000 mL for a primary joint replacement.^[15]^ Relative to the previous point, TKR had a close association with major postoperative blood loss, which amounted in between 800 and 1200 mL; and thus, the requirement for a blood transfusion was frequently necessary.^[16]^ Due to the substantial blood loss, these patients were also in a growing demand for allogeneic blood transfusions, but there were some cases that were followed by some costs and risks, such as longer hospital stays, infectious disease transmission, immunologic reactions, acute lung injury, cardiopulmonary overload, hemolytic, and anaphylactic reactions.^[17]^ Therefore, both the blood loss and the requirement for blood transfusions during TKR must be reduced due to the aforementioned factors. Consequently, we conducted this study in order to investigate the effects rivaroxaban had on both hidden blood loss and BTR following TKR. As a result, we can find a new way to protect patients from the combination of the amount of blood loss and excessive BTR during the TKR process.

## 2 Material and methods

### 2.1 Ethical statement

This study was reviewed and approved by the Ethics Committee of Weifang People's Hospital. Patients and their relatives signed written informed consent in strict accordance with the principle provided.

### 2.2 Study subjects

Our study selected overall 235 KOA patients (males: n = 98; females: n = 137; age: 40–88 years; mean age: 63.8 years) undergoing TKR from December 2011 to January 2015 in Weifang People's Hospital. We used Excel for random grouping, and the specific methods were as follows: the patients were numbered, and the random number was randomly generated by using RNAD() function. According to the mean value of random number, the group is divided into 2 groups: rivaroxaban group and nonanticoagulant group, combined with the experimental design. Inclusion criteria:

1. the age of patients being 18+ years old; and
2. patients with KOA and scheduled to undergo TKR.

Exclusion criteria:

1. patients with abnormal blood coagulation functions prior to surgery and contraindications in association with the requisite anticoagulant therapy;
2. patients with obvious hepatic diseases (such as cirrhosis and chronic active hepatitis);
3. patients who were pregnant or lactating women with positive pregnancy tests;
4. patients who have been taken anticoagulation orally and have been advised by their health care physician to carry out the drug routine; and
5. patients who have received vascular surgery.

### 2.3 Treatment regimens

Tracheal intubation under general anesthesia or combined with a spinal-epidural anesthesia was adopted during the surgery with patients who were placed in the supine position. Median incision of the anterior knee along with the medial parapatellar approach was used as the surgical approach point. During the surgery, patients’ collateral ligaments were routinely loosened and a stabilizing cemented knee joint prosthesis was applied (Zimmer, De Puy, Smith & Nephew). Drainage along with an autologous blood recovery system was applied at the completion point of the surgery. The surgery lasted between 2 and 2.5 h. Following the surgery, an intravenous analgesia was used while a tourniquet was used all throughout the duration of the surgery. Patients involved in the rivaroxaban group received certain anticoagulant regimens: patients began to take rivaroxaban orally about 10 mg/d for 2 weeks within 6 to 8 h following the surgery; anticoagulation therapy was not used in coordination with the nonanticoagulant group after surgery. Patients in both groups began to do ankle flexion and extension, and lower-limb muscle training immediately as anesthesia wore off to test range of motion (ROM) of their previously numbed limbs. The inserted drainage tube was subsequently removed on the second day after surgery with patients receiving some form of joint mobilization or continuous passive motion (CPM) training 2 times a day, 30 min each session. Patients could get out of bed and do some exercises within 5 to 7 days following the surgery and continued to do more knee joint exercise once discharged from the hospital.

### 2.4 Observational indexes

Coagulation function indexes (the number of platelet [PLT], activating both partial thromboplastin time [APTT], and prothrombin time [PT]) were measured 3 days before surgery, as well as on the 4th day after surgery, with changes of each index compared throughout the duration before and after surgery. Hematocrit (Hct) and Ward formula were used in order to calculate the total blood loss.^[18]^ The formula was: total blood loss = preoperative blood volume (PBV)^[19]^ × 2 × (Hct before surgery − Hct after surgery)/(Hct before surgery + Hct after surgery).

The formula for calculating blood volume before surgery is as follows: 
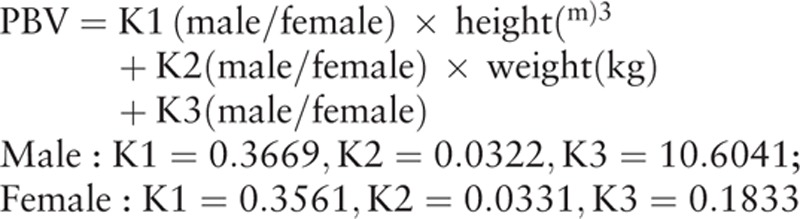


The blood transfusion volume, intraoperative blood loss, postoperative wound drainage, and wound exudates after extubating were recorded (the permeability of each small gauze was 5 mL).

The formula for calculating dominant blood loss was:

Dominant blood loss = intraoperative blood loss + postoperative wound drainage + wound exudates after extubating.

Hidden blood loss = total blood loss after surgery − dominant blood loss.^[20]^

The difference between hemoglobin (HGB) volume before and after surgery was the reduction of HGB. The symptoms including ecchymosis, hematoma and wound infections were recorded. The degree of postoperative pain (visual analogue scale [VAS] scores), the swelling degree of knee joint, and the ROM were also observed. Hospital for special surgery (HSS) scores^[21]^ offered an objective evaluation for the knee joint functions before surgery and at 2 weeks, 3 months, 6 months, 12 months, and 24 months after surgery.

### 2.5 Statistical analysis

The statistical software SPSS 21.0 (IBM Corp., Armonk, NY) was adopted. The measurement data was expressed by adopting the mean ± standard deviation and tested using the *t* test. The enumeration data were expressed by percentage or rate and tested by chi-square test. *P* < .05 was considered as making a statistically significant difference.

## 3 Results

### 3.1 The baseline characteristics of patients

There were 121 and 114 patients involved with the rivaroxaban and nonanticoagulant groups, respectively. No significant differences were found in terms of gender, age, body mass index, height, weight, grading, or course throughout the KOA between the 2 groups (all *P* > .05, Table 1).

**Table 1:**
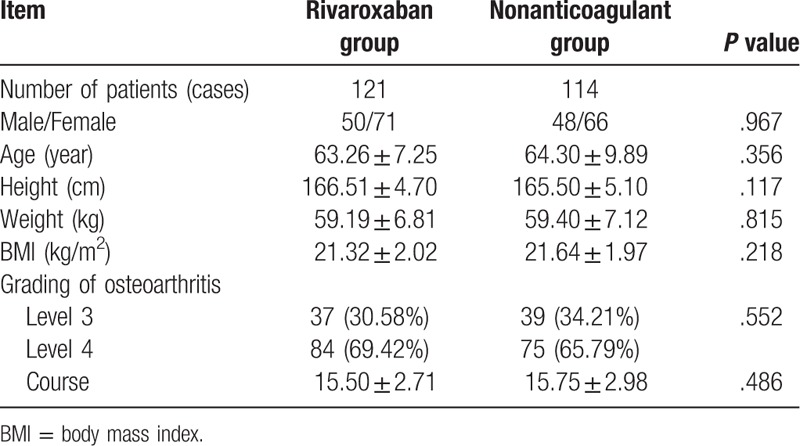
Baseline characteristics of patients in the rivaroxaban and nonanticoagulant groups.

### 3.2 Patients received rivaroxaban regimen has higher hidden blood loss and BTR

In comparison with the nonanticoagulant group, patients apart of the rivaroxaban group had a slightly higher total blood loss concentration, dominant blood loss concentration, and HGB reduction, meanwhile there were no significant differences detected between the 2 groups (both *P* > .05). There was also no significant difference observed among the blood transfusion volume between the 2 groups (*P* > .05). Hidden blood loss concentration of the rivaroxaban group was higher in the rivaroxaban group than that of the nonanticoagulant group (*P* < .05). Among those 121 patients in the rivaroxaban group, 60 patients received blood transfusion with the BTR calculated at 49.59%; while among the 114 patients in the nonanticoagulant group, 40 received blood transfusion with the BTR calculated at 35.09% (*P* < .05) (Table 2).

**Table 2:**
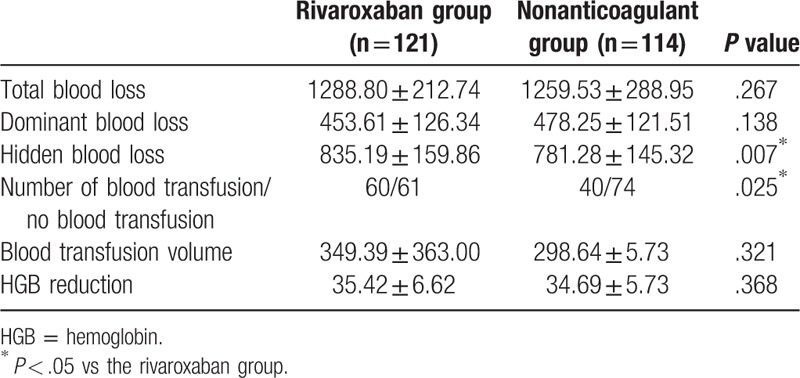
Total blood loss, dominant blood loss, hidden blood loss, blood transfusion volume, and HGB reduction in the rivaroxaban and nonanticoagulant groups.

### 3.3 The coagulation function indexes before and after surgery

Coagulation function indexes of each group before and after surgery were presented in Table 3. Postoperative PLT was higher than the level found in preoperative PLT. The PLT of the rivaroxaban group was slightly lower than that of the nonanticoagulant group, showing no significant difference (*P* > .05). No significant change was also found in the PT before and following surgery. The APTT was also decreased after surgery, but again no significant differences were found in both the PT and APTT between both the rivaroxaban and nonanticoagulant groups (both *P* > .05).

**Table 3:**
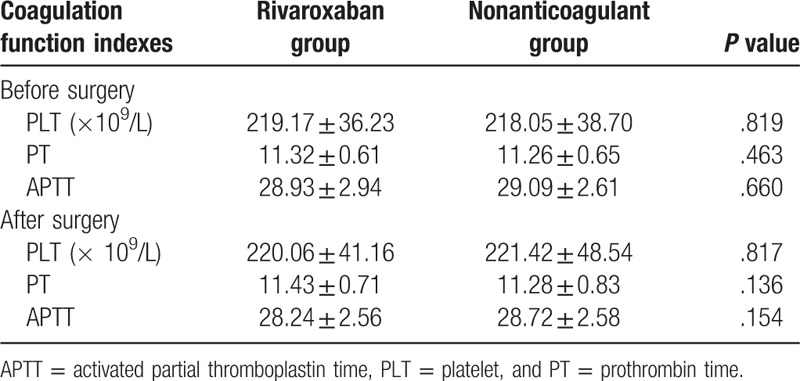
Coagulation function indexes before and after surgery in the rivaroxaban and nonanticoagulant groups.

### 3.4 Rivaroxaban regimen alleviates degree of knee swelling after surgery

The drainage tube was removed on the second day postsurgery with patients receiving some joint mobilization or CPM training 2 times a day, 30 min each session. Patients could excuse themselves from their beds and do some exercises within 5 to 7 days following the surgery. No significant differences were found among the VAS score between the 2 groups at the 3rd and 7th day after surgery (*P* > .05), respectively, in which the use of analgesics after surgery may be one of the reasons for the lack of differences. There were no significant differences in either the passive or active ROM between the 2 groups on both the 3rd and 7th day after surgery (*P* > .05). Compared with the nonanticoagulant group, the degree of knee swelling was lower after surgery in the rivaroxaban group (*P* < .05) (Table 4).

**Table 4:**
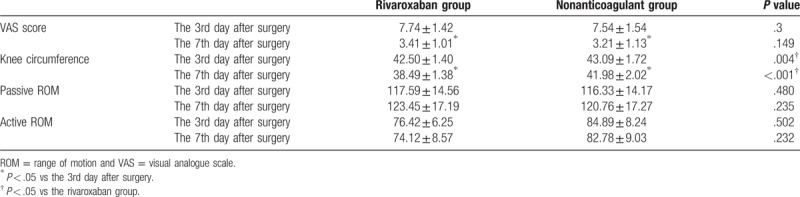
VAS score, degree of knee swelling, and passive and active ROM in the rivaroxaban and nonanticoagulant groups.

### 3.5 Rivaroxaban suppresses the incidence of DVT

No significant difference was again found in the incidence rates of skin ecchymosis around the wound between both the rivaroxaban (50/121, 41.32%) and nonanticoagulant (36/114, 31.58%) groups (both *P* > .05). There were, however, 3 such cases of hematomas in the rivaroxaban group, while the nonanticoagulant group observed 1 case of hematomas; no wound infections occurred in either group. There was also no occurrence of DVT in the rivaroxaban group, while 5 cases were presented in the nonanticoagulant group (*P* *<* .05). No significant differences were found in the knee effusions between both the rivaroxaban (43 cases; 35.54%) and nonanticoagulant (28 cases; 24.56%) groups (*P* > .05). These knee effusions were capable of being self-absorbed after the treatment of hot compression along with physiotherapy. Besides, 2 cases of stress ulcers occurred in the rivaroxaban group at 3 days after surgery, which then presented with symptoms, such as subxiphoid pain and positive fecal hidden blood; however, patients still recovered after the treatment of fasting, gastrointestinal decompression, and gastric mucosal protection. No intracranial hemorrhages or massive hemorrhage of the digestive tract occurred in both groups (Table 5).

**Table 5:**
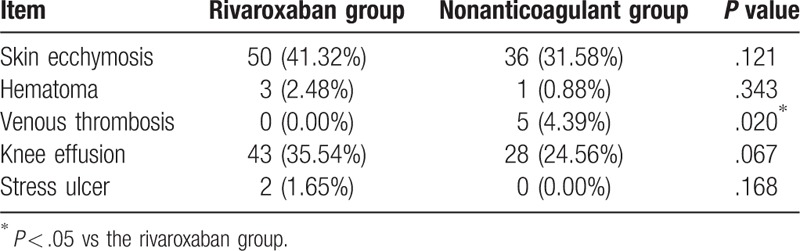
Wound and complications of patients in the rivaroxaban and nonanticoagulant groups.

### 3.6 HSS scores of knee joint functions are higher in patients received rivaroxaban treatment

Significant differences were found in HSS scores of knee joint functions of patients between 2 weeks after surgery and 3 months after surgery (*P* *<* .05). HSS scores of knee joint functions of the rivaroxaban group at 2 weeks following the surgery were obviously higher than those found in the nonanticoagulant group, but no significant differences were found in HSS scores of knee joint functions of patients in both groups at the intervals of 3 months, 6 months, 12 months, and 24 months after surgery (all *P* > .05), which were at the plateau stage (Table 6).

**Table 6:**
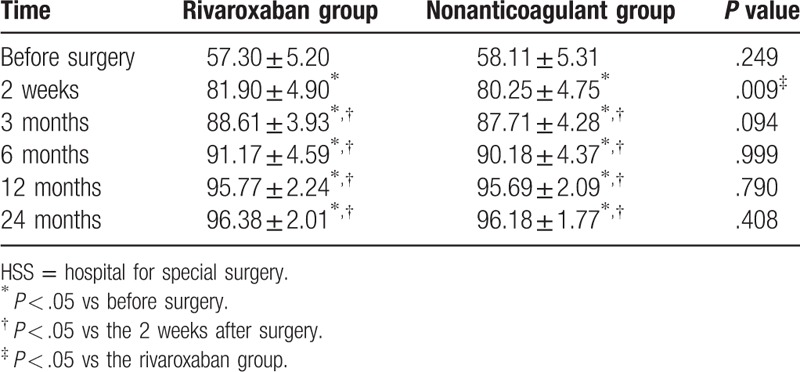
HSS scores of knee joint function of patients before and after surgery in the rivaroxaban and nonanticoagulant groups.

## 4 Discussion

For the elder patients diagnosed with KOA, they were more likely to select TKR as their method of treatment.^[22]^ DVT and PE are fatal perioperative complications after TKR.^[23]^ Selective inhibition of the factor Xa, such as rivaroxaban, could prevent, as well as process DVT, but the risk of wound complications and knee swelling increases with the use of rivaroxaban.^[24,25]^ Therefore, in this present study, we investigated the effects of rivaroxaban on both the hidden blood loss and BTR following TKR. The results showed that hidden blood loss and BTR were both higher, while the degree of the knee swelling was lower following the rivaroxaban therapy, indicating that rivaroxaban could prevent DVT effectively.

Initially, the results indicated that the hidden blood loss and BTR after coagulant therapy had notably increased. As evidenced by a previous study conducted by Sabnis et al, when blood loss occurred after TKR, there was a notable increase in morbidity and hospital stay for the TKR patients.^[26]^ Residual blood in the joint, hemolysis, and tissue extravasation were the forms of hidden blood loss.^[27]^ Due to the tissues bleeding coupled with residual blood in the joint, massive hidden blood loss occurred in patients completing TKR.^[20]^ Patients were frequently required blood transfusion after TKR with transfusion rates ranging from 10% to 38%.^[28]^ The inhibition of rivaroxaban was competitive because it was a highly selective variable for factor Xa.^[29,30]^ Rivaroxaban, similar to factor Xa, played a core role in the coagulation cascade by decreasing thrombin generation.^[31]^ Previous evidence also pointed to the idea that the administration of rivaroxaban resulted in a higher hidden blood loss,^[32]^ which was similarly consistent with our results. Additionally, 1 study focused on the tranexamic acid and rivaroxaban in TKR, finding that patients with the use of rivaroxaban needed more requirement of BTR, compared to those using the tranexamic acid.^[33]^

Second, in comparison with the anticoagulant group, the rivaroxaban group had presented with a lower degree of knee swelling. Additionally, TKR was related to some complications, such as the aforementioned postoperative knee swelling, blood loss, and pain after surgery.^[34]^ Postoperative knee swelling was caused due to many different variables. For example, vascular permeability increased because of the release of both histamine and histamine-like substances, which led to more electrolytes and plasma proteins entering the tissues; and the bleeding caused by the surgery, retained hematoma or infection, and intra-articular bleeding could all contribute to the postoperative knee swelling.^[35,36]^ Moreover, rivaroxaban could make looser plasma clots easily degradable through the fibrinolytic enzymes based on if rivaroxaban was to be put into plasma or whole blood,^[31]^ potentially lowering the degree of knee swelling.

Another point that needed to be emphasized was on the aspects of wound and complications, and our results suggested that rivaroxaban could increase complications and make a positive impact on DVT following TKR. As reflected on in a previous study, the DVT, wound complications, and superficial or deep infection occurred after TKR.^[22]^ Parallel with our study, Wang et al pointed out that the use of rivaroxaban alone in patients with total knee arthroplasty had a higher incidence of bleeding complications.^[37]^ Previous evidence has also demonstrated that rivaroxaban could prevent venous DVT after completion of orthopedic surgery effectively.^[12]^ It had also been reported that the inhibition of prothrombinase activity, as well as the decrease of thrombin generation, were caused due to the factor Xa inhibitor-Rivaroxaban.^[38]^ Fortunately enough, the risk of DVT, nonfatal PE, and death were all reduced by the use of rivaroxaban.^[39,40]^ Besides, for patients undergoing TKR, rivaroxaban was a better, safer, and more effective alternative to enoxaparin in order to prevent VTE,^[38,41]^ consistent with our findings.

In conclusion, our study was meant to demonstrate that anticoagulants had obvious effects on the prevention of DVT following TKR, which provided a useful method for the treatment of patients undergoing a TKR. However, due to the limited sample size in which our study was dealt, further studies are needed in order to increase the reliability of our conclusion; alternative anticoagulant drugs are also necessary in their inclusion to evaluate the optimal outcome of rivaroxaban.

## Acknowledgments

We would also like to thank all participants enrolled in the present study.

## Author contributions

**Conceptualization:** Jian-Yong Liu and Xiu-Li Chen

**Data curation:** Yi-Min Zhang

**Formal analysis:** Miao Zhang and Xiu-Li Chen

**Investigation:** Yi-Min Zhang and Xiao-Guang Liu

**Methodology:** Jian-Yong Liu and Xiao-Guang Liu

**Project administration:** Xue-Dong Sun

**Resources:** Miao Zhang

**Software:** Yi-Min Zhang

**Supervision:** Xue-Dong Sun

**Validation:** Xiao-Guang Liu

**Visualization:** Jian-Yong Liu

**Writing – original draft:** Xue-Dong Sun

**Writing – review and editing:** Miao Zhang and Xiu-Li Chen

## References

[1] Misra D Booth SL Tolstykh I Vitamin K deficiency is associated with incident knee osteoarthritis. Am J Med 2013;126:243–8. 23410565 10.1016/j.amjmed.2012.10.011 PMC3641753

[2] Nicoll D Rowley DI Internal rotational error of the tibial component is a major cause of pain after total knee replacement. J Bone Joint Surg Br 2010;92:1238–44. 20798441 10.1302/0301-620X.92B9.23516

[3] Faldini C Traina F De Fine M Post-operative limb position can influence blood loss and range of motion after total knee arthroplasty: a systematic review. Knee Surg Sports Traumatol Arthrosc 2015;23:852–9. 24682489 10.1007/s00167-013-2732-4

[4] Julin J Jamsen E Puolakka T Younger age increases the risk of early prosthesis failure following primary total knee replacement for osteoarthritis. A follow-up study of 32,019 total knee replacements in the Finnish Arthroplasty Register. Acta Orthop 2010;81:413–9. 20809740 10.3109/17453674.2010.501747 PMC2917562

[5] Chmell MJ Scott RD Total knee arthroplasty in patients with rheumatoid arthritis. An overview. Clin Orthop Relat Res 1999;366:54–60. 10.1097/00003086-199909000-00008 10627718

[6] Kim YH Kulkarni SS Park JW Prevalence of deep vein thrombosis and pulmonary embolism treated with mechanical compression device after total knee arthroplasty in Asian patients. J Arthroplasty 2015;30:1633–7. 25922316 10.1016/j.arth.2015.04.001

[7] Martin MT Nutescu EA Therapeutic potential of apixaban in the prevention of venous thromboembolism in patients undergoing total knee replacement surgery. Curr Med Res Opin 2011;27:2123–31. 21942466 10.1185/03007995.2011.619178

[8] Paul JE Arya A Hurlburt L Femoral nerve block improves analgesia outcomes after total knee arthroplasty: a meta-analysis of randomized controlled trials. Anesthesiology 2010;113:1144–62. 20966667 10.1097/ALN.0b013e3181f4b18

[9] Adam SS McDuffie JR Lachiewicz PF Comparative effectiveness of new oral anticoagulants and standard thromboprophylaxis in patients having total hip or knee replacement: a systematic review. Ann Intern Med 2013;159:275–84. 24026260 10.7326/0003-4819-159-4-201308200-00008

[10] Lindhoff-Last E Samama MM Ortel TL Assays for measuring rivaroxaban: their suitability and limitations. Ther Drug Monit 2010;32:673–9. 20844464 10.1097/FTD.0b013e3181f2f264

[11] Mega JL Braunwald E Wiviott SD Rivaroxaban in patients with a recent acute coronary syndrome. N Engl J Med 2012;366:9–19. 22077192 10.1056/NEJMoa1112277

[12] Landman GW Gans RO Oral rivaroxaban for symptomatic venous thromboembolism. N Engl J Med 2011;364:1178 author reply. 10.1056/NEJMc1100734 21428778

[13] Plosker GL Rivaroxaban: a review of its use in acute coronary syndromes. Drugs 2014;74:451–64. 24535922 10.1007/s40265-014-0188-6

[14] Greiten LE McKellar SH Rysavy J Effectiveness of rivaroxaban for thromboprophylaxis of prosthetic heart valves in a porcine heterotopic valve model. Eur J Cardiothorac Surg 2014;45:914–9. 24306948 10.1093/ejcts/ezt545

[15] Bell TH Berta D Ralley F Factors affecting perioperative blood loss and transfusion rates in primary total joint arthroplasty: a prospective analysis of 1642 patients. Can J Surg 2009;52:295–301. 19680514 PMC2724803

[16] Levy O Martinowitz U Oran A The use of fibrin tissue adhesive to reduce blood loss and the need for blood transfusion after total knee arthroplasty. A prospective, randomized, multicenter study. J Bone Joint Surg Am 1999;81:1580–8. 10565650 10.2106/00004623-199911000-00010

[17] Sizer SC Cherian JJ Elmallah RD Predicting blood loss in total knee and hip arthroplasty. Orthop Clin North Am 2015;46:445–59. 26410634 10.1016/j.ocl.2015.06.002

[18] Gross JB Estimating allowable blood loss: corrected for dilution. Anesthesiology 1983;58:277–80. 6829965 10.1097/00000542-198303000-00016

[19] Nadler SB Hidalgo JH Bloch T Prediction of blood volume in normal human adults. Surgery 1962;51:224–32. 21936146

[20] Sehat KR Evans RL Newman JH Hidden blood loss following hip and knee arthroplasty. Correct management of blood loss should take hidden loss into account. J Bone Joint Surg Br 2004;86:561–5. 15174554

[21] Narin S Unver B Bakirhan S Cross-cultural adaptation, reliability and validity of the Turkish version of the Hospital for Special Surgery (HSS) Knee Score. Acta Orthop Traumatol Turc 2014;48:241–8. 24901911 10.3944/AOTT.2014.3109

[22] Moon HK Han CD Yang IH Factors affecting outcome after total knee arthroplasty in patients with diabetes mellitus. Yonsei Med J 2008;49:129–37. 18306479 10.3349/ymj.2008.49.1.129 PMC2615258

[23] Izumi M Ikeuchi M Aso K Less deep vein thrombosis due to transcutaneous fibular nerve stimulation in total knee arthroplasty: a randomized controlled trial. Knee Surg Sports Traumatol Arthrosc 2015;23:3317–23. 24957913 10.1007/s00167-014-3141-z

[24] Agnelli G Gallus A Goldhaber SZ Treatment of proximal deep-vein thrombosis with the oral direct factor Xa inhibitor rivaroxaban (BAY 59-7939): the ODIXa-DVT (Oral direct factor Xa inhibitor BAY 59-7939 in patients with acute symptomatic deep-vein thrombosis) study. Circulation 2007;116:180–7. 17576867 10.1161/CIRCULATIONAHA.106.668020

[25] Xie J Ma J Huang Q Comparison of enoxaparin and rivaroxaban in balance of anti-fibrinolysis and anticoagulation following primary total knee replacement: a pilot study. Med Sci Monit 2017;23:704–11. 28174415 10.12659/MSM.900059 PMC5312245

[26] Sabnis B Maheshwari R Walmsley P Reducing postoperative blood loss following total hip replacement: a new protocol using rivaroxaban and tranexamic acid. S Afr Orthop Assoc 2013;95:62–4.

[27] Schnurr C Csecsei G Eysel P The effect of computer navigation on blood loss and transfusion rate in TKA. Orthopedics 2010;33:474. 20608630 10.3928/01477447-20100526-08

[28] Hu KZ Sun HY Sui C Effects of five treatment regimens on blood loss and blood transfusion in total knee arthroplasty: a preliminary study in China. Int J Clin Pharmacol Ther 2017;55:433–41. 28139973 10.5414/CP202813

[29] Perzborn E Roehrig S Straub A The discovery and development of rivaroxaban, an oral, direct factor Xa inhibitor. Nat Rev Drug Discov 2011;10:61–75. 21164526 10.1038/nrd3185

[30] Alves C Batel-Marques F Macedo AF Apixaban and rivaroxaban safety after hip and knee arthroplasty: a meta-analysis. J Cardiovasc Pharmacol Ther 2012;17:266–76. 22134134 10.1177/1074248411427402

[31] Varin R Mirshahi S Mirshahi P Whole blood clots are more resistant to lysis than plasma clots--greater efficacy of rivaroxaban. Thromb Res 2013;131:e100–9. 23313382 10.1016/j.thromres.2012.11.029

[32] Zou Y Tian S Wang Y Administering aspirin, rivaroxaban and low-molecular-weight heparin to prevent deep venous thrombosis after total knee arthroplasty. Blood Coagul Fibrinolysis 2014;25:660–4. 24695091 10.1097/MBC.0000000000000121

[33] Wood AM Smith R Keenan A Using a combination of tranexamic acid and rivaroxaban in total knee replacements reduces transfusion requirements: A prospective cohort study. J Arthrosc Jt Surg 2014;1:76–81.

[34] Ishida K Tsumura N Kitagawa A Intra-articular injection of tranexamic acid reduces not only blood loss but also knee joint swelling after total knee arthroplasty. Int Orthop 2011;35:1639–45. 21253725 10.1007/s00264-010-1205-3 PMC3193960

[35] Brock TM Sprowson AP Muller S Short-stretch inelastic compression bandage in knee swelling following total knee arthroplasty study (STICKS): study protocol for a randomised controlled feasibility study. Trials 2015;16:87. 25873152 10.1186/s13063-015-0618-0 PMC4359445

[36] Garrett BR Walters J Knee pain, swelling and stiffness after total knee replacement: a survey of South African knee surgeons. SA Orthop J 2011;9:59–66.

[37] Wang Y Chen T Huang H miR-363-3p inhibits tumor growth by targeting PCNA in lung adenocarcinoma. Oncotarget 2017;8:20133–44. 28423618 10.18632/oncotarget.15448 PMC5386750

[38] Diamantopoulos A Lees M Wells PS Cost-effectiveness of rivaroxaban versus enoxaparin for the prevention of postsurgical venous thromboembolism in Canada. Thromb Haemost 2010;104:760–70. 20806107 10.1160/TH10-01-0071

[39] Lassen MR Ageno W Borris LC Rivaroxaban versus enoxaparin for thromboprophylaxis after total knee arthroplasty. N Engl J Med 2008;358:2776–86. 18579812 10.1056/NEJMoa076016

[40] Beyer-Westendorf J Lutzner J Donath L Efficacy and safety of thromboprophylaxis with low-molecular-weight heparin or rivaroxaban in hip and knee replacement surgery: findings from the ORTHO-TEP registry. Thromb Haemost 2013;109:154–63. 23197272 10.1160/TH12-07-0510

[41] Rathbun S Tafur A Grant R Comparison of methods to determine rivaroxaban anti-factor Xa activity. Thromb Res 2015;135:394–7. 25476589 10.1016/j.thromres.2014.11.017

